# Person Re-Identification with Improved Performance by Incorporating Focal Tversky Loss in AGW Baseline

**DOI:** 10.3390/s22249852

**Published:** 2022-12-15

**Authors:** Shao-Kang Huang, Chen-Chien Hsu, Wei-Yen Wang

**Affiliations:** Department of Electrical Engineering, National Taiwan Normal University, Taipei 106, Taiwan

**Keywords:** person re-identification, AGW baseline, person recognition, multiple object detection, focal Tversky loss

## Abstract

Person re-identification (re-ID) is one of the essential tasks for modern visual intelligent systems to identify a person from images or videos captured at different times, viewpoints, and spatial positions. In fact, it is easy to make an incorrect estimate for person re-ID in the presence of illumination change, low resolution, and pose differences. To provide a robust and accurate prediction, machine learning techniques are extensively used nowadays. However, learning-based approaches often face difficulties in data imbalance and distinguishing a person from others having strong appearance similarity. To improve the overall re-ID performance, false positives and false negatives should be part of the integral factors in the design of the loss function. In this work, we refine the well-known AGW baseline by incorporating a focal Tversky loss to address the data imbalance issue and facilitate the model to learn effectively from the hard examples. Experimental results show that the proposed re-ID method reaches rank-1 accuracy of 96.2% (with mAP: 94.5) and rank-1 accuracy of 93% (with mAP: 91.4) on Market1501 and DukeMTMC datasets, respectively, outperforming the state-of-the-art approaches.

## 1. Introduction

Person re-identification [1,2,3] has become one of the most important computer vision techniques for data retrieval over the past years and is commonly used in vision-based surveillance systems equipped with multiple cameras, each having a unique viewpoint non-overlapped by others [4,5]. When an image is captured by a camera, the person of interest can be located by utilizing object detection methods, such as YOLO [6], RCNN [7], and SSD [8]. Given the person’s image as a query, person re-identification is applied to measure the similarity between the query image and the images in a gallery to generate a similarity ranked list ordered from the highest to the lowest one. To fulfill this task, it is necessary to provide robust modeling for the body appearance of a person of interest rather than relying on biometric cues (human faces) [9]. This is because the captured image may not always be the frontal view of the person. Traditional person re-identification approaches generally focus on gathering information from color [10] and local feature descriptors [11]. However, it is not easy to model complicated scenarios through such low-level features. Thanks to the advance of GPU computational capability and machine learning techniques, the trend of person re-identification has turned into learning-based approaches [2,12,13,14,15,16,17] that can make predictions better than humans [14]. However, existing person re-identification approaches encounter prediction difficulties in different viewpoints, illumination changes, unconstrained poses of the person, poor image quality, appearance similarity among different persons, and occlusion. Hence, person re-ID is still a challenging issue in the field of computer vision.

Typically, person re-ID systems can be separated into three components, including feature representation learning, deep metric learning, and ranking optimization. First, feature representation learning determines the choice of input data format and the architecture design. The former searches for an effective design between using single-modality and heterogeneous data [18,19,20]; the latter [21,22,23,24] focuses on the model backbone construction for generating features that maximize the Euclidian distance of features between different persons and minimizes the distance of features targeting the same person. In the early years, the popular research trend has been to use the global features of the person of interest, such as the ID-discriminative embedding model [25]. Then, several widely-used approaches have proven the benefits of using local features or features from meaningful body parts [12,26,27]. Next, deep metric learning focuses on the training loss design and sampling strategy, which will be introduced in more detail in Section 2.3. Last, ranking optimization dedicates itself to improving the initial ranked list by revising or remapping the similarity scores via various algorithms. Liu et al. [28] proposed a one-shot negative feedback selection strategy to resolve the inherent visual ambiguities. Ma et al. [29] designed an adaptive re-ranking query strategy with respect to the local geometry structure of the data manifold to boost the identification accuracy. Zhong et al. [30] ensured a true match in the initial ranked list according to the similarity of the gallery image and the probe in *k*-reciprocal nearest neighbor. However, the enhancement of ranking optimization highly depends on the quality of the initial ranked list. In other words, an advanced design of feature representation learning and deep metric learning is still needed.

In this work, we aim to enhance the deep metric learning with a more effective design. Inspired by a vital concept of many effective loss designs using a combination of multiple types of loss function, we investigate and decide to incorporate a focal Tversky loss in the AGW [2] baseline. Nevertheless, the support of feature representation and re-ranking are also considered in our re-ID design. Different from the original setting of using ResNet [31] as the model backbone, ResNeSt50 [32] is used in the proposed method to obtain a better feature representation of the person of interest. Besides, a re-ranking technique is also applied to make a final lift in re-ID performance. To this end, the contribution of this work can be summarized as follows:

- We propose a novel training loss design for incorporation into the AGW baseline in the training process to enhance the prediction accuracy of person re-identification. To the best of our knowledge, this work is the first to incorporate a focal Tversky loss in deep metric learning design for person re-identification.
- Different from the original AGW, a re-ranking technique is applied in the proposed method to give a boost to improve the person re-identification performance in the inference mode.
- The proposed method does not require additional training data, and it is easy to implement on ResNet, ResNet-ibn [33], and ResNeSt backbones. Moreover, the proposed method achieves state-of-the-art performance on the well-known person re-identification datasets, Market1501 [34] and DukeMTMC [35]. Besides, we investigate the receiver operating characteristic (ROC) performance among the above three backbones to verify the sensitivity and specificity among various thresholds.

The rest of the paper is organized as follows: Section 2 introduces the related works; Section 3 presents the proposed method; Section 4 shows the training detail and experimental results; Section 5 contains a discussion that highlights the main observations and several open issues for further research, and a conclusion is given in Section 6.

## 2. Related Works

### 2.1. Video-Based Person Re-Identification

Close-world person re-ID can be categorized into video-based and image-based approaches. Video-based person re-ID [36,37,38,39,40] is a re-ID task that uses video sequences as the format of the input query data. Hence, the re-ID model has to learn features that represent multiple frames. The challenge of this approach lies in three parts: (1) accurately capturing temporal information, (2) distinguishing informative frames and filtering out the outlier frames, and (3) handling unconstrained frame quantity. McLaughlin et al. [36] designed an RNN architecture that utilizes both color and optical flow data to obtain appearance and motion information for video re-ID. Chung et al. [37] presented a two-stream Siamese architecture and a corresponding objective training function for learning spatial and temporal information separately. Li et al. [38] proposed a global–local temporal representation that sequentially models both the short-term temporal cues and long-term relations among inconsecutive frames for video re-ID. Hou et al. [39] proposed a network that recovers the appearance of the partially occluded region to obtain more robust re-ID predictions.

### 2.2. Image-Based Person Re-Identification

Compared with the video-based person re-ID, image-based person re-ID approaches have received significant interest over the past years. This is because most of the image-based methods have achieved state-of-the-art performance in closed-world datasets [2]. Popular approaches revealed in recent years include PCB [36], BoT [41], SCSN [42], AGW [2], and FlipReID [43]. Among them, Sun et al. [36] proposed a part-based convolutional baseline (PCB) containing a strategy of uniform partition on convolutional features and a pooling strategy to refine the uniform partition error. Luo et al. [41] introduced plenty of techniques to improve the training process and presented a strong baseline named bag of tricks (BoT). Chen et al. [42] utilized a salience-guided cascaded suppression network (SCSN) that embedded the appearance clues of a pedestrian in a diverse manner and integrated those embedded data into the final representation. Ye et al. [2] improved the BoT method by adding a non-local attention in the ResNet backbone, replacing max pooling with general mean pooling, and using a triplet loss in deep metric learning. Ni and Esa [43] utilized a special flipped structure, named FlipReID, in the training process to narrow the gap of embedded features between the original query image and its horizontally flipped variant. 

### 2.3. Loss Metrics on Person Re-Identification

As far as the design of the deep metric learning of person re-identification is concerned, several popular loss functions are widely used, including identity loss, verification loss, and triplet loss. The identity loss formulates person re-ID into a classification problem. When a query image is fed into the re-ID system, the output of the system is the ID or name of the person of interest. The cross entropy [25] function is widely used to obtain the identity loss. The verification loss seeks an optimal pair-wise solution between two subjects. Widely used functions include contrastive loss [44] and binary verification loss [45]. The former can be represented with a linear combination of a pairwise distance in embedding feature space and a binary label (1/0 indicating true/false match), while the latter one discriminates the positive and negative of image pair sets. Triplet loss [46] treats the re-ID as a clustering task that follows the guideline of controlling the feature distance between the positive and negative pairs. More specifically, the distance between the positive pair should be smaller than the negative pair in a defined margin. We observe that most of the approaches use a combination of the above three kinds of loss. However, to the best of our knowledge, none of the approaches incorporate focal Tversky loss in the training loss design for re-ID. The use of focal Tversky loss to address the problem of data imbalance has been proven effective for networks focusing on learning hard examples during training [47]. Incorporating the focal Tversky loss in deep metric learning can be helpful to leverage the overall performance of person re-ID. Hence, we are motivated to design a novel loss function incorporating the focal Tversky loss.

## 3. Method

**Figure 1:**
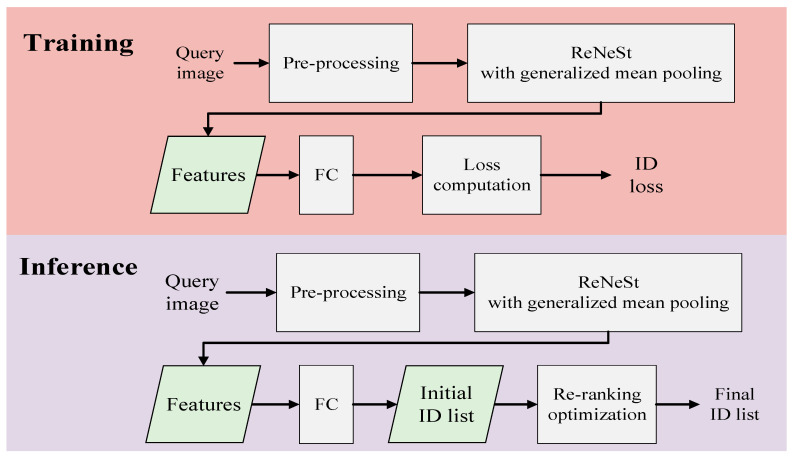
Framework of the proposed method.

The framework of the proposed method is shown in Figure 1. When an input query image is fed into the system, the image is pre-processed and fed into the ResNeSt Backbone structure pre-trained by ImageNet [48]. Then, a loss computation module is introduced to obtain ID loss in the training process. On the other hand, the process in the inference mode is exactly the same, except that a re-ranking optimization is applied after the initial ID list is generated. Note that the proposed method is built on top of the AGW baseline.

### 3.1. Feature Generator

**Figure 2:**
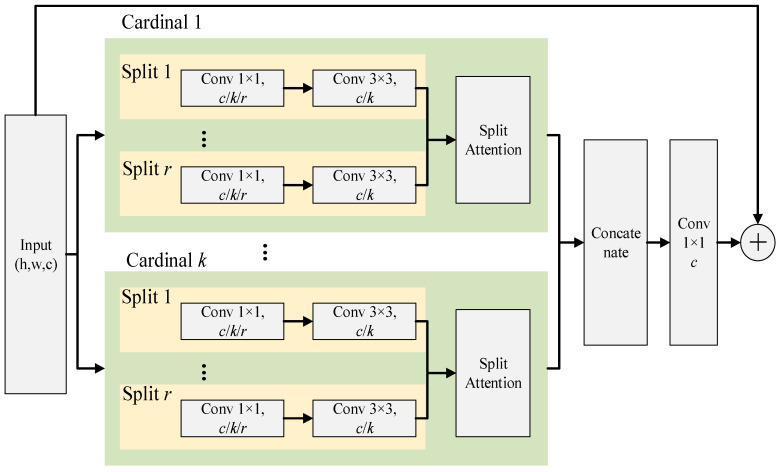
ResNeSt block [32] © 2022 IEEE.

In the pre-processing module, the input image is resized to a uniform scale of 256 × 128 pixels. We then normalize the RGB channel of the image with a mean (0.485, 0.456, 0.406) and standard deviation (0.229, 0.224, 0.225), following the settings of ImageNet [47]. Subsequently, we zero pad 10 pixels on the borders of each image before taking a random crop of size 256 × 128 pixels. After that, these cropped images will be randomly sampled to compose training batches. Different from the AGW baseline, we replace the ResNet50 backbone with the ResNeSt50 backbone, which contains a split attention block as shown in Figure 2. The advantage of using ResNeSt block is that it can extract individual salient attributes and hence provide a better image representation. In the setting of this work, the radix, cardinality, and width attributes of ResNeSt block are set to 2, 1, and 64, respectively. In the final stage, the data will be aggregated by generalized mean pooling (GeM) followed by batch normalization for extracting more domain-specific discriminative features that correspond to the important key points of the input image.

### 3.2. Loss Computation

**Figure 3:**
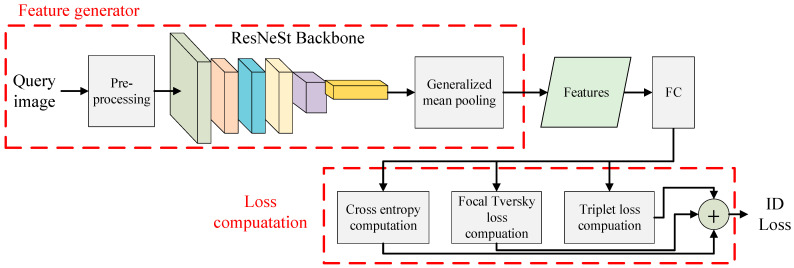
The proposed loss computation.

The proposed loss computation is shown in Figure 3, where the generated features are fed into a fully connected (FC) layer to make ID prediction after the process of the feature generator. The prediction result will then be used to calculate three loss functions, including cross entropy, triplet loss, and focal Tversky loss. While the original AGW only considers the former two loss functions, the proposed method adds a focal Tversky loss that has the advantage of addressing the issue of data imbalance and facilitating the model to learn effectively in a small region of interest [48]. The focal Tversky loss *L_FT_* is defined as:

*L_FT_* = (1 − *L_T_*)^γ^, (1)

where

*L_T_* = TP/(TP + α∙FN + β∙FP). (2)

TP, FN, and FP indicate true positive, false negative, and false positive numbers of the prediction, respectively. α, β, and γ are adjustable parameters. We manually select a set of pre-determined values for the parameters in this work. The final loss design is a combination of the focal Tversky loss, triplet loss, and cross entropy loss:

*L_final_* = *L_FT_* + *L_CE_* + *L_TR_* (3)

### 3.3. Re-Ranking Optimization

**Figure 4:**
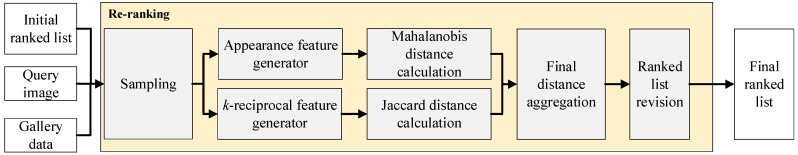
Re-ranking strategy [30] © 2022 IEEE.

In the proposed method, the re-ranking optimization is used in the inference step to enhance the accuracy of the final prediction of person re-identification. Considered as a post-processing tool, the re-ranking with *k*-reciprocal encoding [30] is applied after the initial ID list is generated as shown in Figure 4. The reason for using re-ranking in the proposed method is that it helps to enable a more accurate prediction in person re-ID. Besides, it is acceptable for data retrieval to execute in offline mode. The parameter setting is the same as that of the original paper. As the initial ranked list is generated, the top-*k* samples of the ranked list are encoded as reciprocal neighbor features and utilized to obtain *k*-reciprocal features. Then, the Jaccard distance is calculated with the *k*-reciprocal features of both images. Next, the Manhalanobis distance of feature appearance aggregates with the Jaccard distance to obtain the final distance. Finally, the initial ranked list is revised according to the final distance.

## 4. Experimental Results

To evaluate the proposed person re-ID system, we conducted our experiments on an Intel (R) Core (TM) i7-7700 @ 3.6 GHz and an NVIDIA GeForce RTX 3090 graphic card. The well-known Market1501 and DukeMTMC datasets were used to evaluate the performance of the proposed method against state-of-the-art approaches. Market1501 is a dataset for person re-identification, wherein six cameras were placed in an open-system environment to capture images. It targets 1501 identities and contains a total of 32,668 + 500 K annotated bounding boxes and 3368 query images. DukeMTMC is a dataset focusing on 2700 identities, which contains more than 2 million frames using eight cameras deployed on the Duke University campus. Note that the Adam method was adopted to optimize the model, and the training epoch number was set to 200. The parameters (α, β, and γ) of the focal Tversky loss were manually determined to be (0.7, 0.3, 0.75) and (0.7, 0.3, 0.95) to train on the Market1501 and DukeMTMC datasets, respectively. For easy reference, the hyperparameters of the proposed method are summarized in Table 1.

In the first experiment, we compare the performance of person re-identification with several state-of-the-art approaches, including PCB [36], BoT [41], SCSN [42], AGW [2], and FlipReID [43]. The evaluation metrics are rank-1 accuracy (R1), mean of average precision (mAP), and mean inverse negative penalty (mINP) [2]. The comparison of the re-ID performance is shown in Table 2, where we can see that the proposed method with the ResNeSt50 backbone achieves state-of-the-art performance on both the Market1501 and DukeMTMC datasets. Although the mAP of FlipReID (mAP: 94.7) is slightly higher than that of the proposed method (mAP: 94.5) on the Market1501 dataset, the rank-1 accuracy of the proposed method (R1: 96.2) is superior to that of FlipReID (R1: 95.8). Moreover, compared with FlipReID, our method has the same rank-1 accuracy but higher mAP on the DukeMTMC dataset. Furthermore, we can see that the accuracy of the proposed method without re-ranking is still superior to the original AGW on both of the two datasets. This indicates that applying focal Tversky in deep metric learning does help boosting the prediction accuracy for person re-ID.

Now, there is a question as to whether the validation of the proposed loss design comes directly and entirely from the superior backbone that we have chosen? Hence, it motivates us to investigate whether the loss design is still effective in boosting the person re-identification accuracy on the same backbone as the original AGW holds. We therefore conduct the same experiment on ResNet50 and ResNet50-ibn, and the results are listed in Table 3. We can see from Table 3 that the overall performance of the proposed method is still slightly better than the AGW baseline, even without the re-ranking process. Moreover, on the DukeMTMC dataset, the proposed method with ResNeSt50 backbone still holds first place compared to the other two backbone settings. However, on Market1501, the ResNet50-ibn with re-ranking holds the best performance on rank-1 and mINP. In fact, if the proposed method incorporates the re-ranking technique, the overall performance on Market1501 dataset is similar to each other among the ResNet50, ResNet50-ibn, and ResNeSt50 backbones, because there is no obvious difference between the scores of the proposed method without re-ranking among the three kinds of backbone. In other words, the re-ranking technique results in an almost identical boost in accuracy in person re-identification performance when the initial ranked list is similar. 

**Figure 5:**
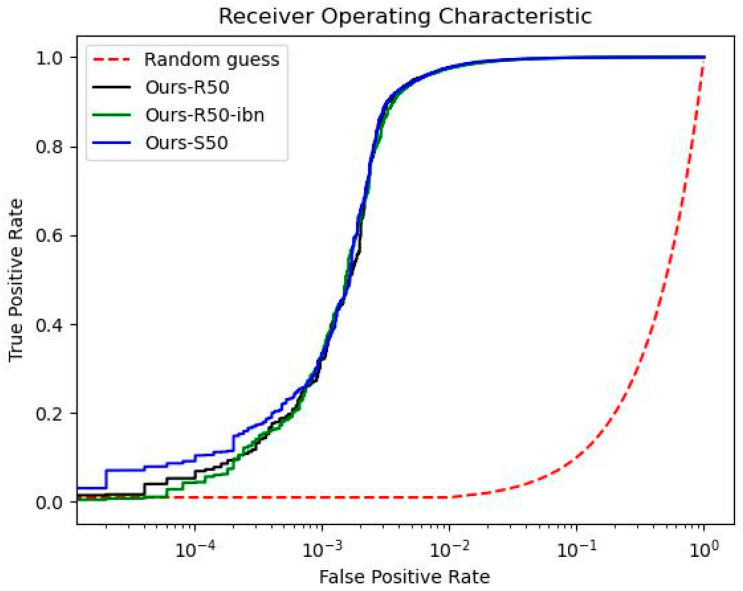
ROC curve with the horizontal axis on a logarithmic scale on Market1501 dataset.

**Figure 6:**
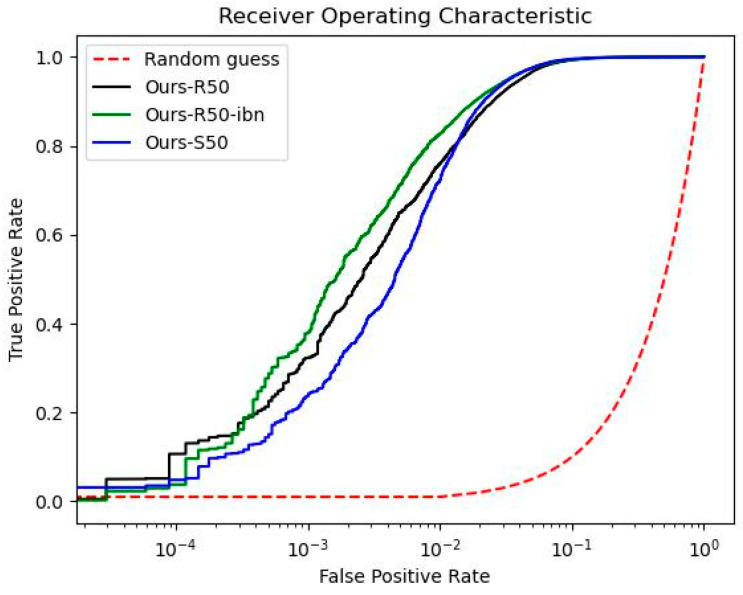
ROC curve with the horizontal axis on a logarithmic scale on DukeMTMC dataset.

The other metric for evaluating the performance of person re-identification is the ROC. As shown in Figure 5 and Figure 6, the vertical axis and horizontal axis of the ROC plot indicate the true positive rate and false positive rate, respectively. In this metric, it shows the classification performance on various threshold settings. The closer the curve becomes to near the top-left corner of the plot, the better the model performs in the prediction of the selected elements. As an attempt to compare the performance among different backbones in the same deep metric learning, we conducted an experiment on the two datasets by the proposed method with the three kinds of backbone settings. The ROC results on Market1501 and DukeMTMC are shown in Figure 5 and Figure 6, respectively. Note that “Ours-R50”,”Ours-R50-ibn”, and “Ours-S50” in the two figures indicate the proposed method using ResNet50, ResNet50-ibn, and ResNeSt50, respectively. Without using a linear scale, the horizontal axis of the ROC is plotted on a logarithmic scale instead for better clarity. In Figure 5, we can see that the three curves almost overlap with each other when the false positive rate is more than 10^−3^. On the other hand, if the false positive rate is less than 10, the model with ResNeSt50 is slightly closer to the top-left corner than the others. In Figure 6, the deviation is more obvious, but the model with ResNet50-ibn outperforms the other two backbones. Besides, the model with ResNeSt50 does not perform well in the ROC test even though it holds the highest accuracy on rank-1, mAP, and mINP metrics in Table 3. Through the above experiments, we have found that the performance of person re-ID in accuracy is not correlated with the performance in sensitivity and specificity.

## 5. Discussion

The experimental results have shown improved performance on the AGW baseline by incorporating focal Tversky loss in the proposed training loss. However, there is still room for improvement in this design. First, parameter tuning is one of the bottlenecks of this method. An optimal design of the three parameters (α, β, and γ) of focal Tversky loss for a particular closed-world dataset is not necessarily optimal in training on other datasets or the same dataset with additional virtual images generated by using data augmentation techniques. Besides, the tuning task demands a computational effort causing an extra cost in applying the method to larger datasets. Next, the re-ranking post-processing design limits the person re-identification method from working in real time. Although the re-ranking method with *k*-reciprocal neighbor used in this work is one of the most widely-used approaches, it is nevertheless challenging to seek an optimal solution that can balance both the accuracy and computational cost in an effective manner. Last, in the investigation of the ROC curve in Figure 5 and Figure 6, we can see that the method with the highest accuracy does not guarantee its performance in sensitivity (true positive rate) and specificity (false positive rate) among various thresholds. This phenomenon indicates that the re-ID model with the highest rank-1 accuracy may not be as accurate as it can be if it is applied to extract features on other datasets. As a result, it has to be carefully examined to apply the trained re-ID model to other open-world datasets. Nevertheless, it is our future research objective to eliminate the above drawbacks for better re-ID performance in terms of accuracy, speed, and robustness. 

## 6. Conclusions

In this work, we have proposed a novel deep metric learning design that incorporates a focal Tversky loss in the AGW baseline and achieves an improved re-ID performance according to the experimental results. Due to the use of focal Tversky loss, the AGW re-ID baseline can address the data imbalance issue and learn effectively on the hard examples in the training process so as to improve the overall person re-ID accuracy. Besides, we have also evaluated the performance of the proposed method on various backbone settings in comparison with the original AGW baseline. Experimental results show that the overall performance of the proposed method is still better than the AGW baseline, even without the re-ranking process. On the other hand, by applying the re-ranking as a post-processing technique, the proposed method outperforms the state-of-the-art methods in rank-1 and mAP metrics on the Market1501 and DukeMTMC datasets. Moreover, an observation of the ROC curve in this work indicates that threshold settings should be carefully examined when applying the re-ID model to extract features, even if the model holds the highest rank-1 accuracy. The insight gained from this investigation is helpful for using the re-ID model as a feature extractor on open-world datasets.

## Figures and Tables

**Table 1:** Hyperparameters of the proposed method.

Hyperparameters	Our Settings
Backbone	ResNeSt50
Optimizer	Adam
Feature dimension	2048
Training epoch	200
Batch size	64
Base learning rate	0.00035
Random erasing augmentation	0.5
Cross entropy loss	(epsilon, scale) = (0.1, 1)
Triplet loss	(margin, scale) = (0, 1)
Focal Tversky loss	Market1501: (α, β, γ) = (0.7, 0.3, 0.75) DukeMTMC: (α, β, γ) = (0.7, 0.3, 0.95)

**Table 2:** Person re-ID performance comparison with state-of-the-art methods on the Market1501 and DukeMTMC datasets (Boldface indicates the best results).

Method	Backbone	Market1501			DukeMTMC		
		R1	mAP	mINP	R1	mAP	mINP
PCB (ECCV2018) [36]	ResNet50	92.3	77.4	-	81.8	66.1	-
BoT (CVPRW 2019) [41]	ResNet50	94.4	86.1	-	87.2	77.0	-
SCSN (CVPR 2020) [42]	ResNet50	95.7	88.5	-	91.0	79.0	-
AGW (TPAMI 2020) [2]	ResNet50	95.1	87.8	65.0	89.0	79.6	45.7
FlipReID (ArXiv 2021) [43]	ResNeSt50	95.8	**94.7**	-	**93.0**	90.7	-
Ours (w/o re-ranking)	ResNeSt50	95.6	89.6	69.5	92.0	82.6	50.2
Ours (with re-ranking)	ResNeSt50	**96.2**	94.5	**88.0**	**93.0**	**91.4**	**77.0**

**Table 3:** Person re-ID performance comparison with various backbone settings on the Market1501 and DukeMTMC datasets (Boldface indicates the best results).

Method	Backbone	Market1501			DukeMTMC		
		R1	mAP	mINP	R1	mAP	mINP
AGW (TPAMI 2020)	ResNet50	95.1	87.8	65.0	89.0	79.6	45.7
Ours (w/o re-ranking)	ResNet50	95.3	89.0	67.8	89.6	80.0	45.9
Ours(with re-ranking)	ResNet50	96.1	**94.7**	88.0	91.1	89.4	73.8
Ours (w/o re-ranking)	ResNet50-ibn	95.6	89.3	68.1	90.3	80.7	47.4
Ours(with re-ranking)	ResNet50-ibn	**96.2**	94.6	**88.1**	92.2	89.9	74
Ours (w/o re-ranking)	ResNeSt50	95.6	89.6	69.5	92.0	82.6	50.2
Ours(with re-ranking)	ResNeSt50	**96.2**	94.5	88.0	**93.0**	**91.4**	**77.0**

## Data Availability

Not applicable.
